# Pharmacogenetics of hepatocellular carcinoma and cholangiocarcinoma

**DOI:** 10.20517/cdr.2019.006

**Published:** 2019-09-19

**Authors:** Marta Alonso-Peña, Anabel Sanchez-Martin, Paula Sanchon-Sanchez, Meraris Soto-Muñiz, Ricardo Espinosa-Escudero, Jose J.G. Marin

**Affiliations:** ^1^Experimental Hepatology and Drug Targeting (HEVEFARM), IBSAL, University of Salamanca, Salamanca 37007, Spain.; ^2^Center for the Study of Liver and Gastrointestinal Diseases (CIBERehd). Carlos III National Institute of Health, Madrid 28029, Spain.

**Keywords:** Anticancer drug, chemoresistance, chemotherapy, cholangiocarcinoma, germline mutation, hepatoma, liver cancer, somatic mutation

## Abstract

Primary liver cancers constitute the fourth most deadly group of cancers. Their poor prognosis is due in part to the pre-existence and/or development, often during treatment, of powerful mechanisms accounting for the poor response of cancer cells to antitumor drugs. These include both impaired gene expression and the appearance of spliced variants, polymorphisms and mutations, affecting the function of genes leading to the reduction in intracellular concentrations of active agents, changes in molecular targets and survival pathways, altered tumor microenvironment and phenotypic transition. The present review summarizes available information regarding the role of germline and somatic mutations affecting drug transporters, enzymes involved in drug metabolism, organelles and signaling molecules related to liver cancer chemoresistance. A more complete picture of the actual complexity of this problem is urgently needed for carrying out further pharmacogenomic studies aimed to improve the management of patients suffering from hepatocellular carcinoma or cholangiocarcinoma.

## Introduction

Primary liver cancers (PLCs) are an important proportion of total malignant neoplasias, constituting the fourth cause of cancer-related death worldwide. According to data from Global Cancer Observatory, there are more than 840,000 new cases of PLCs diagnosed each year and, due to their late diagnosis and poor prognosis, this is accompanied by high mortality, which accounts for approximately 8% of deaths due to cancer.

The most frequent PLC is hepatocellular carcinoma (HCC). This is usually diagnosed by imaging techniques and determination of serum tumor markers, mainly alpha-fetoprotein, followed by confirmatory histological study of the biopsy^[1]^. HCC etiopathogenetic is often difficult to define, with different potentially involved factors, such as genetic alterations (chromosomal and gene mutations), epigenetic changes, and risk factors like cirrhosis, metabolic diseases such as NASH, dietary aflatoxin B1 in Asian countries or viral hepatitis^[2-4]^. The best curative option for early stages is surgical resection, liver transplant or radiofrequency ablation. Unfortunately, HCC is often diagnosed at intermediate or advanced stages. For these patients, the first-line treatment is transarterial chemoembolization (TACE) in the intermediate stage and systemic chemotherapy in the case of advanced HCC^[1,5]^. The response to conventional chemotherapeutic agents, for instance cisplatin, interferon, 5-fluorouracil and doxorubicin in the so-called PIAF regimen, is often very poor due to intrinsic or acquired chemoresistance. Among new targeted drugs, sorafenib, an inhibitor of several tyrosine kinase receptors (TKR), is currently used as the first-line treatment in patients with advanced HCC^[6]^. Nevertheless, the benefit in terms of median overall survival (OS) is only of 2.8 months^[2,5,6]^. Regorafenib, another tyrosine kinase inhibitor (TKI) also approved by FDA, has a similar effect to sorafenib and is now being used as a second-line treatment for patients who cannot tolerate sorafenib treatment or undergo tumor progression during sorafenib therapy^[7]^. Recently, other TKIs have been approved for being used against advanced HCC resistant to sorafenib, such as nivolumab, cabozantinib and lenvatinib^[8]^.

Cholangiocarcinoma (CCA), the second most frequent type of PLC (10%-15% of all PLCs) is a heterogeneous group of malignancies derived from the biliary epithelium. Depending on the anatomical location, CCA is classified into intrahepatic (iCCA), perihilar (pCCA) and distal (dCCA) types. CCA etiopathogenesis has been associated with certain risk factors, such as advanced age, obesity, alcohol consumption, chronic biliary diseases (e.g., primary sclerosing cholangitis and liver cirrhosis), chronic infection by liver flukes (e.g., *Clonorchis sinensis* and *Opisthorchis viverrini*), viral hepatitis, congenital diseases (e.g., Caroli disease), drugs or chemicals (e.g., smoking, thorotrast and dioxin). The diagnosis of CCA is usually based on the combination of imaging techniques, because specific histological and serum biochemical markers are still under investigation^[9,10]^. Surgical resection is the best curative therapy for CCA but this option is only possible in a few cases. For the rest of CCA patients with unresectable or metastatic cancer, conventional systemic chemotherapy (gemcitabine combined with cisplatin as first-line treatment or gemcitabine alone) or locoregional therapy, such as TACE, transarterial radioembolization or radiofrequency ablation, could be an alternative. The use of targeted therapies based on either TKIs, such as erlotinib and lapatinib, or antibodies, such as bevacizumab, cetuximab, and panitimumab has resulted of little benefit^[11]^.

**Figure 1:**
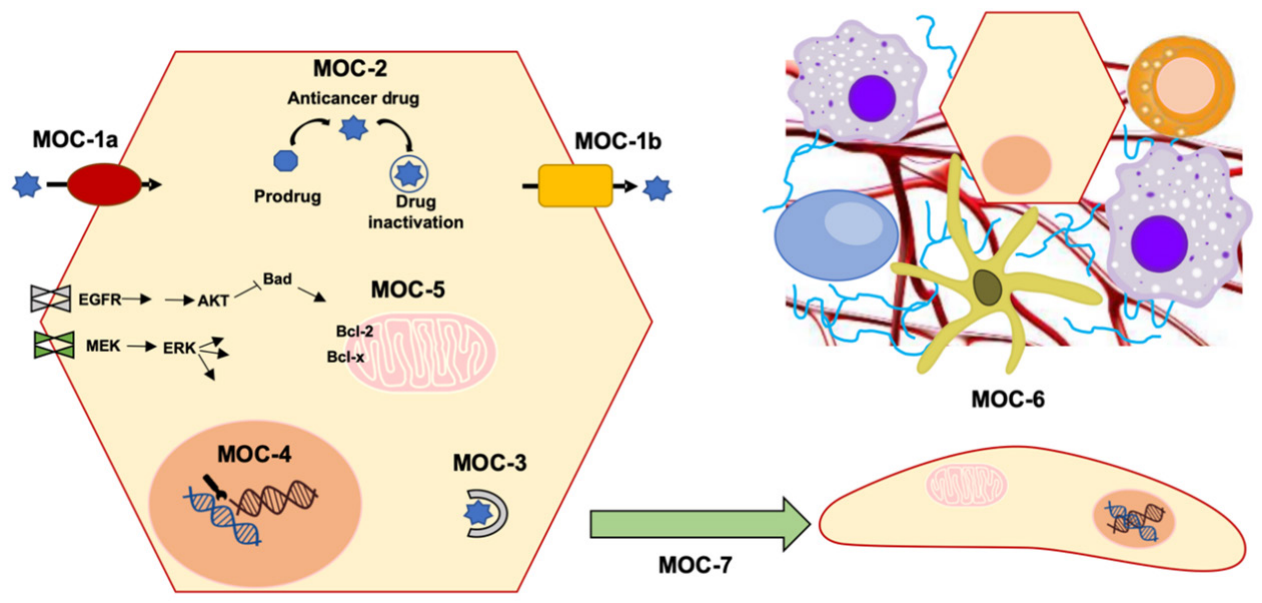
Scheme of mechanisms of chemoresistance (MOC): reduction in intracellular concentration of active drugs (MOC-1 and MOC-2), changes in molecular targets (MOC-3), enhanced DNA repair mechanisms (MOC-4), altered balance between survival and apoptosis pathways (MOC-5), tumor microenvironment (MOC-6) and epithelial-mesenchymal transition (MOC-7)

Despite the efforts in the development of novel treatments to improve PLCs outcome, advances have been modest. One of the most important challenges in PLC pharmacology is to overcome the poor response of these tumors to anticancer drugs, which is due in part to powerful mechanisms of chemoresistance (MOC). These include not only impaired gene expression, but also the existence of genetic variants affecting the function of proteins involved in MOC. Lower intracellular levels of active agents can be mediated by changes in the transportome resulting in impaired drug uptake (MOC-1a), enhanced drug export (MOC-1b), or alterations in drug metabolism that could lead to impaired prodrug activation or increased proportion of inactive metabolites (MOC-2). Additionally, alterations in: i) target genes of antitumor drugs, ii) the activity of mechanisms involved in DNA repair and iii) unbalance between survival and apoptosis factors, are involved in chemoresistance. These processes are classified into MOC-3, MOC-4 and MOC-5, respectively. Finally, the role of changes related to tumor environment (MOC-6) and epithelial-mesenchymal transition (EMT, MOC-7) in PLC chemoresistance is still poorly understood [Figure 1]^[12]^.

**Figure 2:**
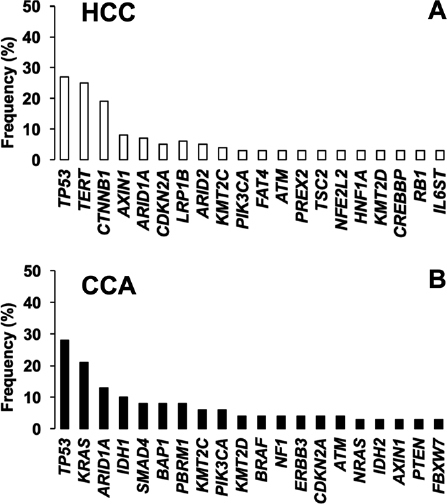
Top 20 most frequently mutated genes in A: hepatocellular carcinoma (HCC); and B: cholangiocarcinoma (CCA). Adapted from COSMIC database (https://cancer.sanger.ac.uk/cosmic)

Given the complexity and heterogeneity of PLCs, the use of personalized diagnosis based on the analysis of genetic variants is becoming an urgent need to establish an optimized treatment for each patient. Therefore, the clinical relevance of pharmacogenetic studies is increasing. The mutational signature has identified the main genes with the most relevant alterations both in HCC and CCA. This includes oncogenes and tumor suppressor genes involved in signaling pathways related to survival, proliferation, differentiation and DNA repair [Figure 2]. In this review, we have summarized current knowledge regarding mutations identified in HCC and CCA, and their role in multidrug resistance (MDR) phenotype and patient outcome. We have distinguished between somatic mutation, i.e., acquired by tumor cells during carcinogenesis, and germline mutations, i.e., inherited genetic alterations. For the nomenclature of the mutations that appear in this review, the updated recommendations of the Sequence Variant Description Working Group^[13]^, which operates under the auspices of three international organizations: the Human Genome Variation Society, the Human Varioma Project and the Human Genome Organization (HUGO), have been followed. Single-nucleotide polymorphisms (SNP) have been considered substitutions of a single nucleotide that occur within a population with a frequency higher than 1%, whereas a single-nucleotide variant, without any limitations of frequency, that may arise in cancer cells is called a single-nucleotide alteration (SNA).

## Changes in intracellular concentrations of active anticancer agents

Many anticancer drugs perform their therapeutic action inside tumor cells. For this reason, mechanisms reducing their intracellular concentrations impair the effectiveness of the treatment. In this sense, changes in the activity of transporters accounting for drug uptake or efflux could determine the capability of anticancer drugs to reach their molecular targets. Moreover, some drugs are administered as prodrugs, which means that they need to be metabolized intracellularly to generate active compounds. In contrast, other drugs are rapidly biotransformed into inactive metabolites. Thus, changes in the expression and activity of drug-metabolizing enzymes can determine the overall response to chemotherapy.

### Mutations affecting the transportome (MOC-1)

Two main superfamilies of transporters are involved in MOC-1: Solute carrier (SLC) proteins and ATP-binding cassette (ABC) proteins. Members of the first group are involved in the uptake of a wide range of molecules, while several ABC pumps use the energy released by the ATP hydrolysis to export their substrates from the cells.

#### Genetic variants in genes involved in drug uptake (MOC-1a)

Among drug uptake transporters, those encoded by *SLCO, SLC22A* and *SLC31A* gene families have been extensively described as main players in the transport of anticancer drugs used against HCC and CCA, such as platinum derivatives and TKIs. Moreover, *SLC28A* and *SLC29A* gene families, which encode transporters able to carry out concentrative (CNT) and equilibrative (ENT) nucleoside uptake, are involved in the response to nucleoside and pyrimidine base analogs, such as gemcitabine and 5-FU^[14]^. Accordingly, mutations affecting these genes could modify the response of HCC and CCA to their substrates. Until now, most investigations have been focused on the association between gene expression and drug resistance. There is also information on the role of germline mutations in antitumor drug pharmacokinetics. In contrast, there is only a few studies regarding somatic mutations affecting SLC transporters in HCC and CCA. Available information can be obtained from COSMIC (https://cancer.sanger.ac.uk/cosmic) and TCGA (https://cancergenome.nih.gov/) databases. Table 1 provides a summary of mutations affecting *SLCO*, *SLC22A*, *SLC28A*, *SLC29A* and SLC31A genes in HCC and CCA.

**Table 1:** Germline (G) and somatic (S) mutations affecting coding (c) and non-coding (nc) regions of SLC genes in primary liver cancer

Gene	Protein	Genetic mutations	G/S	Region	Protein mutations	Functional consequence	Clinical consequences	Studies	References
SLCO1B1	OATP1B1	c.521T>C	G	c	Val174Ala	ND	Thrombocytopenia	HCC patients	[17]
		c.388A>G	G	c	Asn130Asp	ND	Diarrhea	HCC patients	[17]
		c.1039T>G	S	c	Leu347Val	Moderate	ND	TCGA-LIHC	TCGA
		c.152C>A	S	c	Ser51Tyr	Moderate	Pathogenic	TCGA-LIHC	TCGA
SLCO1B3	OATP1B3	c.334T>G	G	c	Ser112Ala	ND	Neutropenia	Unresectable liver metastasis	[18]
		c.699G>A	G	c	Met233Ile	ND	Neutropenia	Unresectable liver metastasis	[18]
		c.391C>A	S	c	Pro131Thr	Moderate	ND	TCGA-LIHC	TCGA
		c.10C>A	S	c	His4Asn	Moderate	ND	TCGA-LIHC	TCGA
		c.166G>A	S	c	Glu56Lys	Moderate	Pathogenic	TCGA-LIHC	TCGA
		c.*12T>C	S	nc	3´ UTR	Modifier	ND	TCGA-LIHC	TCGA
SLC22A1	OCT1	c.1260delGAT	G	c	Met420del	High	Diarrhea	Unresectable liver metastasis	[18]
		c.262T>C	S	c	Cys88Arg	Moderate	Lower sorafenib transport	HCC and CCA patients	[24]
		c.566C>T	S	c	Ser189Leu	Moderate	Lower sorafenib transport	HCC and CCA patients	[24]
		c.659G>T	S	c	Gly220Val	Moderate	Lower sorafenib transport	HCC and CCA patients	[24]
		c.859C>G	S	c	Arg287Gly	Moderate	Lower sorafenib transport	HCC and CCA patients	[24]
		c.262delT	S	c	Cys88Ala fs*16	High	Lower sorafenib transport	HCC and CCA patients	[24]
		c.181delCGinsT	S	c	Arg61Ser fs*10	High	Lower sorafenib transport	HCC and CCA patients	[24]
SLC22A2	OCT2	c.470A>G	S	c	Asn157Ser	Moderate	ND	TCGA-LIHC	TCGA
SLC22A3	OCT3	c.442T>A	S	c	Cys148Ser	Moderate	ND	TCGA-LIHC	TCGA
SLC22A4	OCTN1	c.*34C>A	S	nc	3´ UTR	Modifier	ND	TCGA-LIHC	TCGA
SLC22A5	OCTN2	c.765C>G	S	c	Asp255Glu	Moderate	ND	TCGA-LIHC	TCGA
		c.680G>A	S	c	Arg227His	Moderate	Pathogenic	TCGA-LIHC	TCGA
		c.1564G>A	S	c	Asp522Asn	Moderate	ND	TCGA-CHOL	TCGA
SLC28A1	CNT1	c.461+367T>A	S	nc	Intron	Modifier	ND	TCGA-LIHC	TCGA
		c.461+452G>T	S	nc	Intron	Modifier	ND	TCGA-LIHC	TCGA
SLC28A3	CNT3	c.1105T>C	S	c	Ser369Pro	Moderate	Pathogenic	TCGA-LIHC	TCGA
		c.-26C>T	S	nc	5´ UTR	Modifier	ND	TCGA-LIHC	TCGA
SLC29A1	ENT1	c.149C>A	S	c	Ser50Tyr	Moderate	ND	TCGA-LIHC	TCGA
SLC29A2	ENT2	c.658C>T	S	c	Arg220Cys	Moderate	Pathogenic	TCGA-LIHC	TCGA
		c.-143T>A	S	nc	5´ UTR	Modifier	ND	TCGA-LIHC	TCGA
SLC29A3	ENT3	c.548G>C	S	c	Ser183Thr	Modifier	ND	TCGA-LIHC	TCGA
SLC31A1	CTR1	c.-35-14361C>A	G	nc	Intron	ND	Response to gemcitabine plus platinum treatment	CCA and gallbladder cancer patients	[25]

Data obtained from referred literature and TCGA database. Functional consequences are based on VEP (Variant Effect Predictor; https://www.ensembl.org/vep) impact: High means that the variant is supposed to cause a high disruptive impact on the protein, which is likely to cause loss of function; Moderate means that the variant may be not disruptive, but results in a decrease effectiveness of the encoded protein; Modifier is usually referred to non-coding variants, whose impact is difficult to determine, although they can be involved in transcription or splicing changes. CCA: cholangiocarcinoma; HCC: hepatocellular carcinoma; ND: not described; TCGA: the cancer genome atlas; TCGA-LIHC: the cancer genome atlas - liver hepatocellular carcinoma; TCGA-CHOL: the cancer genome atlas - cholangiocarcinoma

Germline pharmacogenetics: Among the members of *SLCO* gene family, OATP1B1 (*SLCO1B1*) and OATP1B3 (*SLCO1B3*), which have redundant substrate specificity, have been characterized as transporters of TKIs, including sorafenib^[15]^. Several *in vivo* and *in vitro* studies have described SNPs or haplotypes that result in altered expression, localization and activity of OATPs. Most research has been focused on germline polymorphisms of OATP1B1 and OATP1B3 affecting pharmacokinetics and response of statins and paclitaxel, respectively^[16]^. Two germline mutations in OATP1B1, c.388A>G (p.Asn130Asp) and c.521T>C (p.Val174Ala), have been associated with side effects after treatment of HCC patients with sorafenib. However, none of the investigated polymorphisms has been associated with the survival of these patients^[17]^. In patients with unresectable liver metastasis from colorectal cancer, genetic variants of OATP1B3 (c.334T>G; p.Ser112Ala and c.699G>A; p.Met233Ile) and OCT1 (*SLC22A1*, c.1260_1262delGAT; p.Met420del) have been linked to neutropenia and diarrhea, respectively, when they were treated with hepatic artery infusion of irinotecan, oxaliplatin and 5-FU, and intravenous cetuximab^[18]^. Several OCT3 (*SLC22A3*) variants have been studied, but none of them have been related neither to HCC nor to CCA^[19,20]^.

On the other hand, germline mutations in *SLC28A* and *SLC29A* genes affecting gemcitabine effectiveness have been identified in breast cancer^[21]^ and non-small-cell lung cancer^[22,23]^. Unfortunately, there are no similar studies in PLCs.

CTR1 (*SLC31A1*) is a copper transporter involved in the uptake of platinum derivatives. The study of the relationship between CTR1 polymorphisms and the response of CCA to the therapy with gemcitabine plus platinum did not reveal a clear association between SNPs and the treatment outcome, which could be due to the advanced stage of the disease in patients included in the cohort^[25]^. In contrast, the same study proposed that the combination of *SLC31A1* c.-35-14361C>A with other SNP in *ERCC1* (see below) could be a good predictor of the response to gemcitabine plus platinum treatment^[25]^. Furthermore, a significant relationship between two *SLC31A1* intron variants, platinum resistance and clinical outcome has been described in Chinese non-small-cell lung carcinoma patients^[26]^.

Somatic pharmacogenetics: Although downregulation of OATP1B1 and OATP1B3 in HCC, CCA and advanced metastatic liver tumors has been reported^[27]^, no information on somatic mutations affecting these transporters in PLCs is available. Regarding *SLC22A* genes, several variants of OCT1 have been identified in PLCs, including SNAs and splicing variants^[24]^. Among them, several inactivating variants, such as c.262T>C (p.Cys88Arg), c.566C>T (p.Ser189Leu), c.659G>T (p.Gly220Val) and c.859C>G (p.Arg287Gly), were detected with a higher frequency in HCC and CCA than in the adjacent non-tumor tissue. *In vitro* studies showed that these and other OCT1 mutations found in PLCs, such as c.262delT (p.Cys88Alafs*16) and c.181delCGinsT (p.Arg61Serfs*10), result in lower sorafenib uptake and hence poorer induced cytotoxicity. Short non-functional *SLC22A1* mRNA variants have also been detected in other malignancies, such as glioma^[28]^ and chronic myeloid^[29-31]^ and lymphocytic^[32]^ leukemia. Moreover, not only mRNA abundance but also the correct localization of OCT1 at the plasma membrane is important for the response of HCC patients to sorafenib^[33]^. The reduction in *SLC22A1* expression has been associated with advanced tumor stages and shorter survival of patients with HCC^[34]^ or CCA^[35]^.

Evidence for reduced OCT3 expression in HCC and CCA has also been found. *In vitro* experiments in cisplatin resistant hepatoma cells have shown reduced OCT3 expression in these cells, which resulted in lower cisplatin uptake, whereas induced OCT3 overexpression restored the sensitivity of these cells to cisplatin^[36]^. Whether, in addition to changes in transcription, there are associated somatic mutations is not known.

Some studies have described a correlation between low *SLC29A1* expression and poor prognosis in HCC patients^[37]^, whereas up-regulation of *SLC29A2* has been associated with advanced stages, vascular invasion and poor survival in these patients^[38]^. However, no further research on somatic mutations affecting these transporters has been reported.

#### Genetic variants in genes involved in drug export (MOC-1b)

ABC transporters mediate the active efflux of a large variety of compounds, including antitumor drugs. Thus, a high expression/activity of these pumps induces a decrease in intracellular drug concentrations that plays an important role in the MDR phenotype of PLCs^[39]^. Several mutations affecting these transporters may determine the response of HCC and CCA to their substrates [Table 2].

**Table 2:** Germline (G) and somatic (S) mutations affecting coding (c) and non-coding (nc) regions of ABC genes in primary liver cancer

Gene	Protein	Genetic mutations	G/S	Region	Protein mutations	Functional consequences	Clinical consequences	Studies	References
ABCB1	MDR1	c.1537A>T	S	c	Ile513Phe	Moderate	ND	TCGA-LIHC	TCGA
		c.2621T>C	S	c	Val874Ala	Moderate	ND	TCGA-LIHC	TCGA
		c.20G>A	S	c	Arg7His	Moderate	ND	TCGA-LIHC	TCGA
		c.246delA	S	c	Gly83Efs*3	High	ND	TCGA-LIHC	TCGA
		c.466A>T	S	c	Met156Leu	Moderate	ND	TCGA-LIHC	TCGA
		c.1827A>T	S	c	Lys609Asn	Moderate	ND	TCGA-LIHC	TCGA
		c.28887T>G	S	c	Leu963Trp	Moderate	ND	TCGA-LIHC	TCGA
		c.590T>A	S	c	Met197Lys	Moderate	ND	TCGA-LIHC	TCGA
		c.3435C>T	S	c	Ile1145=	ND	Higher risk of HCC recurrence	HCC patients	[50]
ABCC1	MRP1	c.2512A>G	S	c	Ile838Val	Moderate	ND	TCGA-LIHC	TCGA
		c.854C>A	S	c	Pro285Gln	Moderate	ND	TCGA-LIHC	TCGA
		c.2296G>A	S	c	Val766Met	Moderate	ND	TCGA-CHOL	TCGA
		c.2281A>T	S	c	Ile761Phe	Moderate Moderate	ND	TCGA-LIHC	TCGA
		c.2195T>A	S	c	Leu732Gln	Moderate	ND	TCGA-LIHC	TCGA
		c.-1666G>A	S	nc	Promoter	ND	Lower expression	HCC patients	[57]
		c.-260G>C	S	nc	Promoter	ND	Higher expression	HepG2 and Hep3B cells	[58]
ABCC2	MRP2	c.3737T>A	S	c	Leu1246His	Moderate	ND	TCGA-LIHC	TCGA
		c.71C>A	S	c	Pro24Gln	Moderate	ND	TCGA-CHOL	TCGA
		c.1781G>A	S	c	Ser594Asn	Moderate	ND	TCGA-LIHC	TCGA
		c.2810A>G	S	c	Asn937Ser	Moderate	ND	TCGA-LIHC	TCGA
		c.715G>T	S	c	Val239Leu	Moderate	ND	TCGA-LIHC	TCGA
		c.1249G>A	S	c	Val471Ile	ND	Sorafenib efflux	HEK cells	[59]
		c.3972C>T	S	c	Ile1324=	ND	Lower expression	Patients with CCA	[60]
		c.-58A>C	S	nc	5´ UTR	Modifier	ND	TCGA-LIHC	TCGA
		c.-24C>T	S	nc	5’ UTR	ND	Higher expression	Luciferase assay	[61]
ABCC3	MRP3	c.1666_1671dupTACGTG	S	c	Tyr556_Val557	Moderate	ND	TCGA-LIHC	TCGA
		c.614A>C	S	c	Asn205Thr	Moderate	ND	TCGA-LIHC	TCGA
		c.423G>T	S	c	Trp141Cys	Moderate	ND	TCGA-LIHC	TCGA
		c.422G>T	S	c	Trp141Leu	Moderate	ND	TCGA-LIHC	TCGA
		c.800C>A	S	c	Thr267Lys	Moderate	ND	TCGA-LIHC	TCGA
		c.1558G>A	S	c	Gly520Ser	Moderate	ND	TCGA-LIHC	TCGA
		c.2120A>G	S	c	Glu707Gly	Moderate	ND	TCGA-LIHC	TCGA
		c.1936A>C	S	c	Ser646Arg	Moderate	ND	TCGA-LIHC	TCGA
		c.-211C>T	S	nc	5’ UTR	ND	Lower expression Same expression	Healthy liver	[62] [63]
		c.*179-9_*179-7delTCC	S	nc	Intron	Modifier	ND	TCGA-LIHC	TCGA
ABCC4	MRP4	c.1024C>A	S	c	Leu342Ile	Moderate	ND	TCGA-LIHC	TCGA
		c.994G>A	S	c	Val332Met	Moderate	ND	TCGA-LIHC	TCGA
		c.382T>G	S	c	Ser128Ala	Moderate	ND	TCGA-LIHC	TCGA
		c.2174A>T	S	c	Gln725Leu	Moderate	ND	TCGA-LIHC	TCGA
		c.1037T>A	S	c	Ile346Asn	Moderate	ND	TCGA-LIHC	TCGA
		c.1785G>C	S	c	Gln595His	Moderate	ND	TCGA-LIHC	TCGA
ABCC5	MRP5	c.1745A>T	S	c	Asp582Val	Moderate	ND	TCGA-LIHC	TCGA
		c.3724C>T	S	c	Arg1242Cys	Moderate	ND	TCGA-LIHC	TCGA
		c.4145T>C	S	c	Leu1382Phe	Moderate	ND	TCGA-LIHC	TCGA
ABCG2	BCRP	c.34G>A	G	c	Val12Met	ND	Altered sorafenib pharmacokinetics	HCC patients	[43]
		g.89078924T>C	G	c	Intron	ND	Altered sorafenib pharmacokinetics	HCC patients	[43]
		c.734C>G	S	c	Phe245Arg	Moderate	ND	TCGA-LIHC	TCGA
		c.1500G>T	S	c	Lys500Asn	Moderate	ND	TCGA-CHOL	TCGA
		c.745A>G	S	nc	Ile249Val	Moderate	ND	TCGA-LIHC	TCGA
		g.89073197A>G	S	nc	Enhancer region	ND	Lower expression	HepG2 cells	[64]
		g.88924371A>G	S	nc	Enhancer region	ND	Lower expression	HepG2 cells	[64]
		g.89189602G>A	S	nc	Enhancer region	ND	Lower expression	HepG2 cells	[64]
		ABCG2RE1*2	S	nc	Enhancer region	ND	Lower expression	HepG2 cells	[64]
		g.89026428A>C	S	nc	Enhancer region	ND	Higher expression	HepG2 cells	[64]

Data obtained from referred literature and TCGA database. Functional consequences are based on VEP (Variant Effect Predictor; https://www.ensembl.org/vep) impact: High means that the variant is supposed to cause a high disruptive impact in the protein, which is likely to cause loss of function; Moderate means that the variant may be not disruptive, but results in a decrease effectiveness of the encoded protein; Modifier is usually referred to non-coding variants, whose impact is difficult to determine, although they can be involved in transcription or splicing changes. CCA: cholangiocarcinoma; HCC: hepatocellular carcinoma; ND: not described; TCGA: the cancer genome atlas; TCGA-LIHC: the cancer genome atlas - liver hepatocellular carcinoma; TCGA-CHOL: the cancer genome atlas - cholangiocarcinoma

Germline pharmacogenetics: Concerning germline mutations, only those affecting *ABCG2* (c.34G>A; p.Val12Met and the intron variant g.89078924T>C) deserve to be mentioned. Both *in vitro*^[40]^ and *in vivo*^[41]^ studies have demonstrated the ability of the breast cancer resistance protein (BCRP) encoded by *ABCG2* to export sorafenib with higher affinity than MDR1^[42]^. Hence, when present in homozygosis, these mutations have been associated with lower exposure of extratumor tissues and a better response to sorafenib^[43]^.

Somatic pharmacogenetics: MDR1 (*ABCB1*) also known as P-glycoprotein, is involved in the pharmacokinetics of many drugs^[44]^, including sorafenib^[42]^, which is consistent with the fact that MDR1 expression has been inversely correlated with HCC response to pharmacological treatment^[45,46]^. Interestingly, MDR1 has been found highly expressed in CCA biopsies^[47]^ and cell lines^[48]^. Regarding its genetic variability, more than 60 SNAs for *ABCB1* have been described^[49]^. The presence of the synonymous SNP c.3435C>T (p.Ile1145=) in heterozygous patients has been associated with increased levels of MDR1 and higher risk of HCC recurrence^[50]^. This mutation has also been related to a lower exposure to sorafenib in HCC patients^[43,51]^.

Proteins encoded by *ABCC* genes, also known as multidrug resistance-associated proteins, are involved in PLC chemoresistance^[52-56]^. The presence of the polymorphism c.-1666G>A in MRP1 (*ABCC1*) has been correlated with low promoter transcriptional activity^[57]^. The opposite occurs in the case of the variant c.-260G>C^[58]^. Moreover, poor outcome and shorter survival have been described in patients with PLC carrying the c.-1666G>A variant^[57]^.

The best known MRP2 (*ABCC2*) variants are c.-24C>T, c.1249G>A (p.Val471Ile) and c.3972C>T (p.Ile1324=). These frequent variants have been associated with higher chemoresistance and reduced survival rate in many different tumors, including HCC and CCA^[61,65-67]^. Some combinations of these variants in homozygosis are more sensitive to miR-379-induced *ABCC2* mRNA down-regulation, leading to lower MRP2 expression^[68]^. Moreover, expression of the c.1249G>A variant has been associated to enhanced MRP2-mediated sorafenib efflux^[59,69]^.

Owing to its high expression levels, MRP3 (*ABCC3*) plays a key role in the MDR phenotype of CCA^[48,70]^ and is also involved in the poor response of HCC to sorafenib^[71]^. The SNP c.-211C>T, which is also present in healthy liver, alters *ABCC3* promoter activity although its functional repercussion is controversial^[62,63]^. Regarding MRP4 (*ABCC4*) and MRP5 (*ABCC5*), some polymorphisms that modify their stability and substrate specificity have been described^[72-74]^. Nevertheless, their relationship with drug resistance in PLCs remains unknown.

A role of BCRP in HCC chemoresistance has been reported^[75]^, whereas this is not clearly elucidated in the case of CCA^[76]^. In healthy liver tissue, the expression of c.421C>A (p.Gln141Lys) variant correlates with low BCRP protein levels^[77]^. In addition, several SNPs that modify enhancer activity at the *ABCG2* gene locus have been reported^[64]^. Four of these variants (g.89073197A>G, g.88924371A>G, g.89189602G>A and ABCG2RE1*2, which is a combination of g.88923906G>A, g.88924176C>T and g.88924371A>G) decreased the promoter activity and hence reduced gene expression, contrary to g.89026428A>C that is associated with increased BCRP activity. Moreover, other genomic variants (g.89073197A>G and g.88924371A>G) increase the ability of *ABCG2* gene to bind to nuclear proteins in human hepatoma HepG2 cells^[64]^.

### Mutations affecting drug metabolism (MOC-2)

Changes in drug metabolism, either by reduction in the activation of prodrugs or increased inactivation of active agents, can contribute to chemoresistance. The enzymes involved in MOC-2 participate in either phase I reactions (oxidoreduction of substrates) or in phase II (conjugation with polyatomic groups) processes^[39]^. As many anticancer agents are administered as prodrugs, they require metabolic activation by phase I enzymes. Thus, the presence of variants in genes encoding these enzymes is relevant in cancer therapy, because they may reduce the efficacy of several antitumor drugs and increase their adverse effects^[78]^ . In addition, inactivation by phase II enzymes of anticancer drugs, such as TKIs, is an important systemic and intratumor mechanism involved in determining the response to pharmacological treatment^[80]^. Available information regarding the presence of germline and somatic mutations in PLC affecting genes encoding phase I and II enzymes is summarized in Tables 3 and 4, respectively.

**Table 3:** Germline (G) and somatic (S) mutations affecting coding (c) and non-coding (nc) regions of genes coding phase I enzyme in primary liver cancer

Gene	Protein	Genetic mutations	G/S	Region	Protein mutations	Functional consequences	Clinical consequences	Studies	References
DPYD	DPD	c.1700G >T	S	c	Gly567Val	Moderate	Pathogenic	TCGA-LIHC	TCGA
		c.589C>T	S	c	Pro197Ser	Moderate	Pathogenic	TCGA-LIHC	TCGA
		c.491A>C	S	c	Lys164Thr	Moderate	ND	TCGA-LIHC	TCGA
		c.*102A>C	S	nc	3’ UTR	Modifier	ND	TCGA-LIHC	TCGA
		c.483+820G>C	S	nc	Intron	Modifier	ND	TCGA-LIHC	TCGA
DPYS	DHP	c.650A>T	S	c	His217Leu	Moderate	ND	TCGA-LIHC	TCGA
CYP2D6	CYP2D6	c.100C>T	S	c	Pro34Ser	High	Increased HCC susceptibility	Cirrhotic / Fibrotic HCC patients	[79]
CYP2C9	CYP2C9	c.1075A>C	S	c	Ile359Leu	High	ND	Cirrhotic / Fibrotic HCC patients	[79]
CYP2A6	CYP2A6	c.715C>G	S	c	Gln239Glu	Moderate	ND	TCGA-LIHC	TCGA
		c.323A>G	S	c	Asp108Gly	Moderate	Neutral	TCGA-LIHC	TCGA
		c.*527C>G	S	nc	3’ UTR	Modifier	ND	TCGA-LIHC	TCGA
		c.*135A>G	S	nc	3’ UTR	Modifier	ND	TCGA-LIHC	TCGA
		c.194+409A>G	S		Intron	Modifier	ND	TCGA-LIHC	TCGA
CYP3A4	CYP3A4	c.-59A>G	S	nc	5´ UTR	Modifier	ND	TCGA-LIHC	TCGA
CES2	CES	c.278C>G	S	c	Ser93*	High	ND	TCGA-LIHC	TCGA
		c.1524G>A	S	c	Trp508*	High	Neutral	TCGA-LIHC	TCGA
		c.153G>T	S	c	Gln51His	Moderate	ND	TCGA-LIHC	TCGA
EH	EH	c.337T>C	S	c	Tyr113His	Low	Increase risk of HCC	HCC patients	[81]
		c.416A>G	S	c	His139Arg	High	ND	HCC patients	[81]
NQO1	NQO1	c.127T>G	S	c	Tyr43Asp	Moderate	ND	TCGA-LIHC	TCGA

Data obtained from TCGA database and referred literature. Functional consequences are based on VEP (Variant Effect Predictor; https://www.ensembl.org/vep) impact: High means that the variant is supposed to cause a high disruptive impact in the protein, which is likely to cause loss of function; Moderate means that the variant may be not disruptive, but results in a decrease effectiveness of the encoded protein; Low means that the variant has low probability to cause a disruptive change in the encoded protein; Modifier is usually referred to non-coding variants, whose impact is difficult to determine, although they can be involved in transcription or splicing changes. HCC: hepatocellular carcinoma; ND: not described; TCGA: the cancer genome atlas; TCGA-LIHC: the cancer genome atlas - liver hepatocellular carcinoma

**Table 4:** Germline (G) and somatic (S) mutations affecting coding (c) and non-coding (nc) regions in genes coding phase II enzymes in primary liver cancer

Gene	Protein	Genetic mutations	G/S	Region	Protein mutations	Functional consequences	Clinical consecuences	Studies	References
DCK	DCK	c.*823C>T	S	nc	3’ UTR	Modifier	ND	TCGA-LIHC	TCGA
		c.*157G>T	S	nc	3’ UTR	Modifier	ND	TCGA-LIHC	TCGA
CDA	CDA	c.208G>A	G	c	Ala70Thr	High	Neutropenia and decreased clearance of gemcitabine	Several types of cancer	[87]
		c.271A>G	S	c	Met91Val	Moderate	Neutral	TCGA-LIHC	TCGA
		c.267-1G>A	S	c	Splice acceptor	High	Pathogenic	TCGA-LIHC	TCGA
		c.157T>C	S	c	Cys53Arg	Moderate	ND	TCGA-LIHC	TCGA
MET	MET	c.65G>T	S	c	Ser22Ile	Moderate	ND	TCGA-LIHC	TCGA
		c.3713A>T	S	c	His1238Leu	Moderate	ND	TCGA-LIHC	TCGA
		c.3767A>T	S	c	His1256Leu	Moderate	ND	TCGA-LIHC	TCGA
SULT1A1	SULT1A1	c.-265_-258delGTGAGGGG	S	nc	5’ UTR	Modifier	ND	TCGA-CHOL	TCGA
		c.-4-460_-4-453delGTGAGGGG	S	nc	Intron	Modifier	ND	TCGA-CHOL	TCGA
UGT2B7	UGT2B7	c.311C>A	S	c	Thr104Lys	Moderate	Neutral	TCGA-LIHC	TCGA
		c.22G>T	S	c	Val8Leu	Moderate	ND	TCGA-LIHC	TCGA
		c.282_283delTA	S	c	Lys95Glufs*26	High	ND	TCGA-LIHC	TCGA
		c.589_591delGTT	S	c	Val197del	Moderate	ND	TCGA-LIHC	TCGA
UGT1A1	UGT1A1	c.725T>A	S	c	Val242Glu	Moderate	ND	TCGA-LIHC	TCGA
UGT1A3	UGT1A3	c.779A>G	S	c	Asp260Gly	Moderate	ND	TCGA-LIHC	TCGA
		c.457C>T	S	c	Pro153Ser	Modifier	ND	TCGA-LIHC	TCGA
		c.867+13031C>T	S	c	Intron	Modifier	ND	TCGA-LIHC	TCGA
		c.867+17971A>G	S	c	Intron	Moderate	ND	TCGA-LIHC	TCGA
UGT1A9	UGT1A9	c.668T>A	S	c	Phe223Tyr	Moderate	Neutral	TCGA-LIHC	TCGA

Data obtained from TCGA database (https://cancergenome.nih.gov/) and referred literature. Functional consequences are based on VEP (Variant Effect Predictor; https://www.ensembl.org/vep) impact: High means that the variant is supposed to cause a high disruptive impact in the protein, which is likely to cause loss of function; Moderate means that the variant may be not disruptive, but results in a decrease effectiveness of the encoded protein; Modifier is usually referred to non-coding variants, whose impact is difficult to determine, although they can be involved in transcription or splicing changes. ND: not described; TCGA: the cancer genome atlas; TCGA-LIHC: the cancer genome atlas - liver hepatocellular carcinoma; TCGA-CHOL: the cancer genome atlas - cholangiocarcinoma

#### Phase I enzymes

Somatic pharmacogenetics: Cytochrome P450 (CYP) includes a large group of enzymes located in mitochondrial membranes or in the endoplasmic reticulum that play a crucial role in metabolism^[82]^. In humans, the most important CYPs regarding drug metabolism are CYP1A2, CYP2A6, CYP2B6, CYP2C6, CYP2D6, CYP2E6, CYP2C8, CYP2C9 and CYP3A4/5, which are responsible for 90% of the metabolic inactivation of drugs currently used^[83]^. CYPs are abundantly expressed in HCC, which is consistent with the fact that drugs are more rapidly metabolized in the tumor than in the surrounding liver tissue^[84]^. Therefore, changes in CYP activity can contribute to HCC chemoresistance^[85]^. For instance, *CYP2A6* activates the prodrug tegafur/uracil to 5-FU. An investigation on polymorphisms affecting CYP2A6 in Japanese patients with HCC has reported a frequency of 0.233 for the CYP2A6*4 genetic variant, which results in CYP2A6 gene deletion, in heterozygosis, whereas the homozygous genotype was found in 5 out of 58 HCC patients^[86]^. Other study has described that the allelic frequency of the mutant homozygote CYP2D6 c.100C>T (p.Pro34Ser) variant is significantly reduced in HCC patients^[79]^. The authors reported an increased intrinsic clearance of drugs, such as linifanib (ABT-869) and banoxantrone (AQ4N), when the CYP2C9 variant c.1075A>C (p.Ile359Leu) was expressed in HCC^[79]^. CYP3A4 is the major enzyme involved in metabolism of drugs, which includes sorafenib, gefitinib and paclitaxel. However, CYP3A4 is usually very poorly expressed in tumors and cell lines of different origin^[88]^. Thus, CYP3A4 activity has been found markedly decreased in tumors of 96 patients with HBV-positive HCC, as compared with the adjacent non-tumor tissue^[85]^.

Epoxide hydrolase (EH) metabolizes epoxy eicosatrienoinc acids (EETs) and other lipid epoxides and is involved in a variety of biological activities, such angiogenesis and cancer metastasis^[89]^. The microsomal form of EH (mEH) has been characterized and two SNPs in the coding region, c.337T>C (p.Tyr113His) and c.416A>G (p.His139Arg) have been identified. Both variants have lower enzyme activity compared to the wild-type protein^[90,91]^. The relationship between these variants and HCC is poorly understood^[92]^. In a meta-analysis involving 1,696 HCC cases, the His113-mEH allele was significantly associated with increased risk of HCC, whereas the Arg139-mEH genotype had no association with HCC development^[80]^.

Dihydropyrimidine dehydrogenase (DPD, gene symbol *DPYD*), which is highly expressed in human liver, is involved in the first step of pyrimidines breakdown. DPD converts thymine to 5,6-dihydrothymine and uracil to 5,6-dihydrouracil. Accordingly, this catalytic activity can modify the effectiveness of 5-FU^[93]^. Thus, intratumor levels of this drug can vary among patients, despite of receiving the same dose^[94]^. DPD polymorphisms play a key role in this differential response^[86]^. Although more than 200 polymorphisms have been identified, *in vitro* studies have shown that only few of them have a deleterious impact on DPD enzymatic activity^[95]^. Mutations related to this gene are described in Table 3. A second enzyme involved in 5-FU catabolism is dihydropyrimidinase (DHP gene symbol *DPYS*), which catalyzes the conversion of dihydro-5,6-fluorouracil to fluoro-β-ureidopropionate. DHP deficiency caused by heterozygous missense and nonsense polymorphisms in *DPYS* gene may increase 5-FU toxicity^[96]^.

NAD(P)H quinone oxidoreductase 1 (NQO1) catalyzes the reduction of quinones and nitro derivatives using NADP or NADPH as cofactors. NQO1 expression leads to a favorable position for the development of tumor cells by protecting them from oxidative stress and chemotherapeutic agents, resulting in cancer progression and chemoresistance, as has been described for HCC^[97]^. In CCA, NQO1 plays a role in modulating sensitivity of cancer cells to gemcitabine when given in combination with dicoumarol, which enhances gemcitabine cytotoxicity in CCA cells with high NQO1 activity^[98]^. The most prominent and frequent variant of NQO1 is c.609C>T (p.Pro187Ser), which has been associated to an increased risk of colorectal cancer and colorectal adenoma^[99]^ and poor OS in non-small-cell lung cancer^[100]^.

#### Phase II enzymes

Germline pharmacogenetics: Cytidine deaminase (CDA) is the major enzyme of gemcitabine inactivation. This enzyme catalyzes the irreversible hydrolytic deamination of cytidine and deoxycytidine to uridine and deoxyuridine, respectively. CDA, which is poorly expressed in liver tissue^[101]^, presents several SNPs that have been associated with higher expression and enzymatic activity of CDA and poorer disease outcome in patients treated with gemcitabine. Among the most studied variants are two non-synonymous SNPs, c.79A>C (p.Lys27Gln) and c.208G>A (p.Ala70Thr), and three SNPs in the *CDA* promoter region that possibly affect CDA expression, c.-451G>A, c.-92A>G and c.-31delC. Another well-studied variant is the synonymous SNP c.435C>T (p.Thr145=), located at exon 4^[102]^.

Somatic pharmacogenetics: The somatic mutation c.208G>A (p.Ala70Thr) decreases the activity of CDA in pancreas, lung and mesothelium cancer, which has clinical impact in patients treated with gemcitabine, cisplatin and 5-FU^[87,91]^. Moreover, c.208G>A has been associated with a reduced clearance of gemcitabine and increased neutropenia when patients were co-treated with gemcitabine and 5-FU or platinum-containing drugs^[87]^. The impact of c.79A>C and c.435C>T in the clinical outcome of 126 advanced non-small-cell lung cancer patients treated with gemcitabine–platinum-regimens has been evaluated^[103]^. The results indicated that patients with the AC genotype had significantly longer time to progression and OS than patients with CC genotype.

Deoxycytidine kinase (DCK) catalyzes the first rate-limiting phosphorylation step in the activation of deoxycytidine analogs. The combination of three mutations, c.511G>A (p.Glu171Lys), c.739G>A, (p.Glu247Lys) and c.745G>A (p.Leu249Met) in DCK sensitizes a panel of cancer cell lines to treatment with gemcitabine^[104]^.

Several SNPs have been suggested to affect glutathione S-transferases function and favor carcinogenesis. The SNP c.-67C>T in the *GSTA1* promoter, when expressed in hepatocytes, reduces *GSTA1* expression. Moreover, the TT genotype is more frequent in HCC than in healthy controls. In addition, GSTA1 expression is lower in HCC than in healthy livers^[105]^.

Sulfotransferases (SULT) catalyze the addition of a sulfonate moiety. Three human SULT families have been identified: SULT1, SULT2 and SULT4^[106]^. SULT1A1 metabolizes brivanib, a drug used in phase III trials as the first-line treatment of HCC^[107]^. SULT1A1 is up-regulated in patients with HCC secondary to chronic HBV infection^[108]^. Table 4 shows the mutations in SULT1A1 observed in PLC. In order to elucidate the role of these mutations in the chemoresistance of these tumors, further investigations are required.

Uridine 5’-diphospho glucuronosyl transferases (UGT) are a group of phase II drug-metabolizing enzymes that catalyze the glucuronidation of xenobiotics and endogenous compounds^[39]^. A reduction in the activity of UGT1As and UGT2B7 has been observed in HBV-positive HCC^[109]^. In addition, down-regulation of UGT1A9 has been related to lower sorafenib metabolism in microsomes of HCC cells^[110]^. UGT2B7 is a p53 target gene in liver cells that could promote intratumor or systemic metabolism and clearance of cytotoxic agents and other drugs administered together. Thus, UGT2B7 may be related to reduced efficacy of cancer therapy^[111]^. A novel class of human UGT isoforms, namely i2s, has been described. In comparison to isoforms 1 (i1s), i2s isoforms utilize the shorter exon 5b instead of incorporating the usual C-terminus exon 5a, which causes a premature arrest of translation and subsequent loss of the transmembrane domain. Therefore, UGT i2s isoforms are located at the lumen and cytoplasm rather than at the membrane of the endoplasmic reticulum, which results in the lack of glucuronidation activity but acting in a dominant-negative manner. Increased i2 isoforms expression in PLCs has been found^[112]^. Somatic mutations affecting UGTs described in TCGA database are listed in Table 4.

## Molecular targets and survival pathways

Three major types of molecular alterations have been reported to be at the origin of hepatocarcinogenesis: i) Aberrant cell proliferation and survival due to a constitutive activation of signaling pathways, such as EGFR-Ras-MAPK, PI3K-AKT-mTOR, HGF/MET, Wnt-β-catenin and others; ii) Deregulation of proapoptotic machinery elements, such as p53 and Bcl2; and iii) Stimulation of neo-angiogenesis, which is crucial for tumor development^[2]^. Mutations in genes involved in these pathways are expected to determine the response to drugs acting on these targets.

### Molecular targets of chemotherapeutic agents (MOC-3)

Mutations or changes in the expression levels of target genes could prevent efficient drug-target interaction leading to treatment failure^[113]^. Although TKIs are useful in the treatment of many tumors, their efficacy is often hampered by changes in their targets. For instance, the multikinase inhibitor sorafenib reduces tumor cell proliferation and angiogenesis in HCC, which is due in part to its interaction with receptors for several growth factors, such as EGF (EGFR), VEGF (VEGFR) and PDGF (PDGFR)^[6]^.

#### Germline pharmacogenetics

Although somatic mutations are the most frequent changes among the targets of antitumor drugs, some target genes belonging to the VEGF family are also affected by germline mutations. This is the case of *KDR* gene (also known as *VEGFR* or *VEGFR2*), in which the germline SNP c.1416A>T (p.Gln472His), has been described in an East Asian HCC cohort. In this case, patients with two wild-type alleles and heterozygous (AT) genotype have decreased progression-free survival (PFS) and OS compared with homozygous patients for the mutant allele (TT)^[114]^. This polymorphism has also been associated with toxicity and adverse reactions to sorafenib, including increasing risk of hypertension and hand-foot skin reactions in TT patients^[115]^. Moreover, this mutation has been linked to the response to capecitabine/oxaliplatin and cyclophosphamide in colorectal^[116]^ and prostatic^[117]^ tumors, respectively. In addition, the germline polymorphism c.-94C>G at the 5’UTR region of the *VEGFA* gene has been associated with the outcome of prostatic and colorectal cancer patients^[116]^. In HCC, homozygous genotype for the G allele has been related to lower PFS and OS than homozygous patients for C allele and heterozygous genotypes^[118]^.

#### Somatic pharmacogenetics

Acquired resistance to TKI treatment can be due to somatic mutations in a wide variety of target genes. Exome sequencing analysis of 243 HCCs revealed 161 mutated genes which could be classified into 11 recurrent pathways. The most frequently altered pathways were PI3K-AKT-mTOR (51%) and MAPK (43%). Although target genes of TKIs (*EGFR, VEGFR1, KDR, VEGFC, VEGFA* and *BRAF*) were affected by less than 1% of all mutations, these alterations were predicted to have functional consequences^[119]^. Table 5 summarizes mutations described in HCC and CCA. Some of these mutations, for instance affecting EGFR, VEGFR1 and VEGFC, are predicted to alter the function of these proteins^[119]^. An *EGFR* polymorphism, c.2369C>T (p.Thr790Met), has been described in non-small-cell lung cancer, and prevents gefitinib- and erlotinib-induced TKR inhibition^[124,125]^. Somatic mutations in *ESR1*, *TYMS* and *EGFR* genes related to drug resistance have also been reported in PLC^[91]^. A variant in an intron of *VEGFC* (g.177608775T>C) has been associated with sorafenib efficacy in HCC patients. CC genotype of this mutation is accompanied by a decrease in PFS and OS as compared with patients bearing CT or TT genotype^[116]^. In iCCA, EGFR amplification has been associated with the response to gefitinib (anti-EGFR therapy)^[126]^.

**Table 5:** Germline (G) and somatic (S) mutations affecting coding (c) and non-coding (nc) regions in target genes of anticancer drugs in primary liver cancer

Gene	Protein	Genetic mutation	G/S	Region	Protein mutation	Functional consequences	Clinical consequences	Studies	Ref.
BRAF	BRAF	c.1799A>T	S	c	Val600Glu	Moderate	Decreased OS	CCA patients	[120]
							ND	Biliary Adenoma	[121]
		c.1910T>A	S	c	Val637Glu	Activation of MAPK and AKT pathways	Enhanced proliferation	HCC *in vivo*	[122,123]
EGFR	EGFR	c.2464G>A	S	c	Ala822Pro	Moderate	ND	HCC patients	[119]
		c.67C>T	S	c	Arg23Trp	ND	Benign	HCC patients	[119]
		c.374A>G	S	c	Tyr125Cys	ND	ND	HCC patients	dbEMT
		c.2165_2173 dupCCAGCGTGG	S	c	Ala722_Val724dup	Moderate	ND	TCGA-LIHC	TCGA
		c.2095A>G	S	c	Ile699Val	Moderate	Pathogenic	TCGA-LIHC	TCGA
		c.3313A>T	S	c	Thr1105Ser	Moderate	Neutral	TCGA-LIHC	TCGA
		c.1097C>G	S	c	Pro366Arg	Moderate	Pathogenic	TCGA-LIHC	TCGA
		c.926_945 delCGAATATTA AACACTTCAAA	S	c	Thr309fs*17	High	ND	TCGA-LIHC	TCGA
		c.3349A>T	S	c	Ser1117Cys	Moderate	ND	TCGA-LIHC	TCGA
		c.1881-2577C>T	S	nc	Intron	Modifier	ND	TCGA-LIHC	TCGA
		c.1072+33G>T	S	nc	Intron	Modifier	No significant	TCGA-LIHC	TCGA
FLT1	VEGFR1	c.2306G>A	S	c	Ala769Val	Moderate	ND	HCC patients	[119]
		c.2196_2198delTGA	S	c	Ser733*	High	ND	HCC patients	[119]
		c.2110C>T	S	c	Glu704Lys	Moderate	ND	HCC patients	[119]
		c.1796C>G	S	c	Thr599Arg	Moderate	Pathogenic	TCGA-LIHC	TCGA
		c.2021delG	S	c	Ser674fs*12	Modifier	ND	TCGA-LIHC	TCGA
		c.166dupG	S	c	Glu56fs*5	High	ND	TCGA-CHOL	TCGA
		c.1988A>C	S	c	Lys663Thr	Modifier	ND	TCGA-LIHC	TCGA
		c.679A>T	S	c	Asn227Tyr	Moderate	Pathogenic	TCGA-LIHC	TCGA
		c.1997A>T	S	c	Asn666Ile	Moderate	ND	TCGA-LIHC	TCGA
		c.3636-1G>C	S	nc	Splice acceptor	High	Pathogenic	TCGA-LIHC	TCGA
KDR	VEGFR2	c.1416A>T	G	c	Gln472His	ND	Increased PFS and OS	HCC patients	[114]
		c.713A>G	S	c	Val238Ala	ND	Benign	HCC patients	[119]
		c.2935G>A	S	c	Glu979Lys	Moderate	ND	TCGA-CHOL	TCGA
		c.1054G>T	S	c	Ala352Ser	Moderate	Pathogenic	TCGA-LIHC	TCGA
		c.1772T>G	S	c	Leu591Arg	Moderate	Neutral	TCGA-LIHC	TCGA
		c.3944A>G	S	c	Asp1315Gly	Moderate	Pathogenic	TCGA-LIHC	TCGA
		c.1297G>T	S	c	Asp433Tyr	Moderate	ND	TCGA-LIHC	TCGA
		c.3957C>A	S	c	Tyr1319*	High	Pathogenic	TCGA-LIHC	TCGA
		c.3152G>A	S	c	Arg1051Gln	Moderate	Pathogenic	TCGA-CHOL	TCGA
		c.1368C>G	S	c	Ile456Met	Moderate	Pathogenic	TCGA-LIHC	TCGA
		c.2398G>C	S	c	Gly800Arg	Moderate	Pathogenic	TCGA-LIHC	TCGA
		c.*172G>A	S	nc	3´UTR	Modifier	ND	TCGA-LIHC	TCGA
VEGFA	VEGFA	c.-94C>G	G	nc	5’UTR	ND	Decreased PFS and OS	HCC patients	[118]
		c.332_346del GCCCGGGCC TCGGGC	S	c	Ala112_Gly116del	Moderate	ND	TCGA-LIHC	TCGA
		c.*285A>G	S	nc	3´UTR	Modifier	ND	TCGA-CHOL	TCGA
		c.308+1G>C	S	nc	Splice donor	High	ND	TCGA-LIHC	TCGA
VEGFC	VEGFC	c.986C>T	S	c	Gly329Glu	Moderate	ND	HCC patients	[119]
		c.367C>A	S	c	Asp123Tyr	Moderate	ND	HCC patients	[119]
		c.235T>C	S	c	Lys79Glu	Moderate	ND	HCC patients	[119]
		c.842G>A	S	c	Gly281Glu	Moderate	ND	TCGA-LIHC	TCGA
		c.938A>G	S	c	Asn313Ser	Moderate	ND	TCGA-LIHC	TCGA
		c.341A>T	S	c	Tyr114Phe	Moderate	ND	TCGA-LIHC	TCGA
		c.1037C>G	S	c	Thr346Ser	Moderate	ND	TCGA-LIHC	TCGA
		c.1253T>G	S	c	Met418Arg	Moderate	ND	TCGA-LIHC	TCGA
		c.820G>C	S	c	Asp274His	Moderate	ND	TCGA-CHOL	TCGA
		c.-17C>A	S	nc	5’UTR	Modifier	ND	TCGA-LIHC	TCGA
		g.177608775T>C	S	nc	Intron	ND	Decreased PFS and OS	HCC patients	[118]

Data obtained from referred literature, dbEMT, and TCGA database. Functional consequences are based on VEP (Variant Effect Predictor; https://www.ensembl.org/vep) impact: High means that the variant is supposed to cause a high disruptive impact in the protein, which is likely to cause loss of function; Moderate means that the variant may be not disruptive, but results in a decrease effectiveness of the encoded protein; Modifier is usually referred to non-coding variants, whose impact is difficult to determine, although they can be involved in transcription or splicing changes. OS: overall survival; PFS: progression-free survival; CCA: cholangiocarcinoma; HCC: hepatocellular carcinoma; IHCA: Inflammatory hepatocellular adenomas; ND: not described; TCGA: the cancer genome atlas; TCGA-LIHC: the cancer genome atlas - liver hepatocellular carcinoma; TCGA-CHOL: the cancer genome atlas - cholangiocarcinoma

Concerning BRAF, which is another major target of sorafenib, the missense mutation c.1799T>A (p.Val600Glu) must be highlighted. This mutation has been found in many malignant tumors, such as melanoma, thyroid cancer^[123]^, colorectal cancer^[127]^, but also HCC^[119]^ and iCCA^[120]^. In patients with iCCA, OS was lower in those with mutated BRAF (7.4% of cases) than in wild-type cases. The murine ortholog of this mutation in mouse (c.1910T>A; p.Val637Glu), is a frequent feature in mouse liver cancer. In diethylnitrosamine-induced mouse hepatocarcinogenesis, c.1910T>A mutation correlated with Erk1/Akt hyperphosphorylation, suggesting an activation of MAPK and AKT pathways that results in stimulated cell proliferation^[120,123]^. Nevertheless, a relationship between these mutations and the response to TKIs has not been well characterized.

### DNA repair mechanisms (MOC-4)

Cancer cells can repair genome perturbations that are induced by antitumor-drugs through diverse mechanisms that depend on the type of damage suffered by DNA^[113]^. DNA repairing machinery includes direct reversal of lesions by enzymes, such as O-6-methylguanine-DNA methyltransferase (MGMT), nucleotide and base excision repair (NER and BER, respectively), DNA mismatch repair (MMR), homologous recombination (HR) and non-homologous end joining (NHEJ). Deregulated expression and the appearance of mutations in genes of the repair machinery have been observed in a variety of tumors. Since many cytotoxic drugs used in the treatment of PLC act through alterations in DNA structure of cancer cells, MOC-4 play an important role in the response of these tumors to chemotherapy. Table 6 provides a summary of both germline and somatic mutations affecting DNA repair genes in HCC and CCA.

**Table 6:** Germline (G) and somatic (S) mutations affecting coding (c) and non-coding (nc) regions of repair genes in primary liver cancer

Gene	Protein	Genetic mutation	G/S	Region	Protein mutation	Functional consequences	Clinical consequences	Studies	References
APEX1	APE1	c.444T>G	G	c	Asp148Glu	ND	Cisplatin resistance	HCC patients	[131]
BRCA1	BRCA1	c.185delT	G	c		High	Better OS. Therapy response	CCA patients	[132]
		c.5503C>T	S	c	Arg1835*	Moderate	Better OS. Therapy response	CCA patients	[132]
		c.1961delA	S	c	Lys654fs*47	ND	Better OS. Therapy response	CCA patients	[132]
		c.5153G>T	S	c	Trp1718Leu	Moderate	Better OS. Therapy response	CCA patients	[132]
		c.2293G>A	S	c	Glu765Lys	ND	Better OS. Therapy response	CCA patients	[132]
			S	c	Asp825fs*21	ND	Better OS. Therapy response	CCA patients	[132]
BRCA2	BRCA2	c. 6503delT	G	c		High	Better OS. Therapy response	CCA patients	[132]
		c. 6174delT	G	c		High	Better OS. Therapy response	CCA patients	[132]
		c.9976A>T	S	c	Lys3326*	Moderate	Better OS. Therapy response	CCA patients	[132]
			S	c	Leu2368fs*8	ND	Better OS. Therapy response	CCA patients	[132]
			S	c	Asn991fs*3	ND	Better OS. Therapy response	CCA patients	[132]
		c.9154C>T	S	c	Arg3052Trp	Moderate	Better OS. Therapy response	CCA patients	[132]
		c.9257G>C	S	c	Gly3086Ala	ND	Better OS. Therapy response	CCA patients	[132]
ERCC1	ERCC1	c.133A>G	S	c	Ser45Gly	ND	ND	HCC patients	cBioportal
		c.43G>T	S	c	Gly15Trp	ND	ND	HCC patients	cBioportal
ERCC2/XPD	ERCC2/XPD	c.1450A>G	S	c	Thr484Ala	ND	ND	HCC patients	cBioportal
		c.215A>T	S	c	Tyr72Phe	ND	ND	HCC patients	cBioportal
		c.1853T>G	S	c	Val618Gly	ND	ND	HCC patients	cBioportal
		c.1378A>G	S	c	Thr460Ala	ND	ND	HCC patients	cBioportal
NHEJ1	NHEJ1/XLF	c.518C>T	S	c	Thr173Met	ND	ND	HCC patients	cBioportal
XRCC1	XRCC1	c.580C>T	G	c	Arg194Trp	ND	Cisplatin resistance	HCC patients	[131]

Data obtained from cBioportal and referred literature. Functional consequences are based on VEP (Variant Effect Predictor; https://www.ensembl.org/vep) impact: High means that the variant is supposed to cause a high disruptive impact in the protein, which is likely to cause loss of function; Moderate means that the variant may be not disruptive, but results in a decrease effectiveness of the encoded protein; Modifier is usually referred to non-coding variants, whose impact is difficult to determine, although they can be involved in transcription or splicing changes. CCA: cholangiocarcinoma; HCC: hepatocellular carcinoma; OS: overall survival; ND: not determined

#### Germline pharmacogenetics

NER is the most important pathway involved in the elimination of bulky adducts induced by UV irradiation and alkylating agents, such as platinum derivatives. More than 25 polypeptides participate in NER^[128]^. Germline variants in NER elements have been found in several cancers and some studies have related these alterations to the lack of response to platinum-based chemotherapy^[129,130]^. However, mutations in these genes are rarely found in HCC.

BER pathway also plays an essential role in DNA damage repair induced by alkylating agents and irradiation. APE1 is an endonuclease involved in this process that recognizes and cleaves abasic (apurinic/apyrimidinic) sites, where XRCC1 forms a complex with a DNA ligase to repair the gaps that have resulted from base excision. In HCC patients, two genetic polymorphisms in *XRCC1* (c.580C>T; p.Arg194Trp) and *APE1* (c.444T>G; p.Asp148Glu) have been associated with resistance to cisplatin^[131]^.

Germline mutations in several genes belonging to DNA repair pathways are more common in CCA. Variants in *BRCA* and *RAD51* genes (HR pathway) and in *MHL1* and *MSH2* genes (MRR repair pathway) have been found in 11% of CCA analyzed^[133]^, although a relationship between these mutations and treatment response or OS has been rarely reported. In a multicenter retrospective study of CCA patients, improved OS in patients harboring pathogenic BRCA1/2 mutations treated with platinum-based therapy and/or PARP inhibitors (PARPi) have been described^[132]^. This suggests that CCA patients could benefit from targeted therapy, such as PARPi administration, as occurs in other BRCA-associated tumors^[134]^.

#### Somatic pharmacogenetics

Somatic mutations in NER genes are rarely found in HCC. *ERCC1*, one of the key components in this repair mechanism, is mutated at low frequency (< 1%). In a cohort of 372 HCC samples collected by TCGA only two *ERCC1* mutations (c.133A>G; p.Ser45Gly and c.43G>T; p.Gly15Trp) were found in two tumors, even though the functional consequences are unknown. However, this gene is frequently overexpressed in HCC tumors, being associated with cisplatin resistance^[135]^. Another essential NER protein, responsible for DNA damage recognition, is XPC, which is also overexpressed in HCC and could be related to chemoresistance^[136]^. Nevertheless, XPC mutations with clinical relevance have not been reported in HCC. The *XPD* (or *ERCC2*) gene encodes a DNA helicase also involved in this pathway. Four non-synonymous mutations were found in *XPD* in the TCGA HCC cohort. The biological effect of these mutations and their impact on HCC patients regarding their response to chemotherapeutic drugs and OS is not known. However, in bladder cancer, non-synonymous mutations in *XPD* have been associated with sensitivity to cisplatin^[137]^.

Mechanisms involved in the repairing of double-strand breaks, such as HR and NHEJ, are also important in the response to anticancer drugs^[138]^. XRCC4-like factor (*XLF*) is a core member of NHEJ pathway required for the double stranded end joining. Somatic mutations in *XLF* gene occur at a very low frequency in HCC tumors. However, both *in vitro* and *in vivo* experiments have demonstrated that *XLF* knockdown confers sensitivity to drug chemotherapy, suggesting that XLF-mediated increase in NHEJ activity can play a role among mechanisms of chemoresistance in HCC^[139]^.

The frequency of somatic mutations in DNA repair genes with clinical impact in CCA is unknown. Nevertheless, the multicenter retrospective study of CCA patients mentioned above also reported enhanced OS of CCA patients harboring somatic mutations suspected to be pathogenic in BRCA1/2 when treated with platinum-based therapy^[132]^

### Survival pathways and apoptosis (MOC-5)

Most pharmacological regimens currently used in the clinical treatment of cancer are based on the activation of apoptosis in cancer cells. Therefore, impairment of the involved machinery not only results in an uncontrolled cell growth, but also confers resistance to chemotherapy. The lack of response to anticancer drugs may be caused by deregulated expression and the appearance of loss-of-function mutations in pro-apoptotic factors (MOC-5a) or be due to an aberrant activation of anti-apoptotic proteins (MOC-5b)^[39]^. Somatic mutations affecting MOC-5a and MOC-5b genes in PLC are listed in Tables 7 and 8, respectively.

**Table 7:** Somatic (S) mutations affecting coding (c) and non-coding (nc) regions of pro-apoptotic genes in primary liver cancer

Gene	Protein	Genetic mutation	G/S	Region	Protein mutation	Functional consequences	Clinical consequences	Studies	References
CASP8	Caspase-8	c.1225_1226 delTG	S	c	Val410Phefs*28.	Loss of function	Probably chemoresistance	HCC patients	[154]
CDKN2A	CDKN2A	c.248G>A	S	c	His83Tyr	Moderate	Poor prognosis	HCC patients	[119]
		g.21971148_ 21971155del	S	c	Ala68Glufs*49	High	Poor prognosis	HCC patients	[119]
		c.263C>A	S	c	Glu88*	High	Poor prognosis	HCC patients	[119]
		g.21974672del	S	c	Gly52Valfs*77	High	Poor prognosis	HCC patients	[119]
		g.21974711_ 21974728del	S	c	Glu33_Asn 39delinsAsp	High	Poor prognosis	HCC patients	[119]
		c.72C>G	S	c	Arg24Pro	Moderate	Poor prognosis	HCC patients	[119]
		c.36G>T	S	c	Ser12*	High	Poor prognosis	HCC patients	[119]
RB1	RB1	c.381A>T	S	c	Ser127_splice	ND	Early recurrence after resection	HCC patients	[155]
		c.508G>T	S	c	Glu170*	Loss of function	Early recurrence after resection	HCC patients	[155]
		c.646delT	S	c	Phe216fs	ND	Early recurrence after resection	HCC patients	[155]
		c.763C>T	S	c	Arg255*	Loss of function	Early recurrence after resection	HCC patients	[155]
		c.979A>T	S	c	Lys327*	Loss of function	Early recurrence after resection	HCC patients	[155]
		c.1421G>A	S	c	Ser474Asn	ND	Early recurrence after resection	HCC patients	[155]
		c.1472T>C	S	c	Leu491Pro	ND	Early recurrence after resection	HCC patients	[155]
		c.1654C>T	S	c	Arg552*	Loss of function	Early recurrence after resection	HCC patients	[155]
		c.2120delC	S	c	Ser707fs	ND	Early recurrence after resection	HCC patients	[155]
TP53	p53	c.747G>T	S	c	Arg249Ser	Loss of function	Poor prognosis	HCC patients	[143]
		c.469G>T	S	c	Val157Phe	Loss of function	Poor prognosis	HCC patients	[143]
		c.743G>A	S	c	Arg248Gln	Loss of function	Doxorubicin resistance	HCC in vitro	[144]
			S	c	1-132del (truncated variant Δ133p53)	Dominant negative	Poor outcome, 5-FU resistance	CCA patients	[150] [151]
TP63	p63		S	c	1-62del (truncated variant ΔNp63)	Gain of function (antiapoptotic-effect)	Doxorubicin and mitoxantrone resistance. Shorter OS	HCC patients	[147] [148]
TP73	p73		S	c	1-72del (truncated variant ΔNp73)	Gain of function (antiapoptotic-effect)	Shorter OS	HCC patients	[147]

Data obtained from cBioportal database and referred literature. Functional consequences are based on VEP (Variant Effect Predictor; https://www.ensembl.org/vep) impact: High means that the variant is supposed to cause a high disruptive impact in the protein, which is likely to cause loss of function; Moderate means that the variant may be not disruptive, but results in a decrease effectiveness of the encoded protein; Modifier is usually referred to non-coding variants, whose impact is difficult to determine, although they can be involved in transcription or splicing changes. CCA: cholangiocarcinoma; HCC: hepatocellular carcinoma; 5-FU: 5-fluorouracil; OS: overall survival; ND: not determined

**Table 8:** Somatic (S) mutations affecting coding (c) and non-coding (nc) regions of anti-apoptotic genes in primary liver cancer

Gene	Protein	Genetic mutations	G/S	Region	Protein mutation	Functional consequences	Clinical consequences	Studies	References
CTNNB1	Catenine beta-1	c.95A>G/T	S	c	Asp32Gly/Val	Gain-of-function	Controversial	HCC patients	[157,158]
		c.94G>T			Asp32Tyr	ND	ND	HCC patients	[159,160]
		c.94G>C			Asp32His				dbEMT
		c.98C>G/A/T/c.97T>C/G	S	c	Ser33Cys/Tyr/Phe/Pro/Ala	Gain-of-function	Controversial	HCC patients	[157-160]
		c.99_113del15	S	c	Gly34_Gly38delGlyIle HisSerGly	ND	ND	HCC	dbEMT
		c.1202T>A			Leu401His	ND	ND	HCC	dbEMT
		c.110C>G/A/T /c.109T>C/G	S	c	Ser37Cys/Tyr/Phe/Pro/Ala	Gain-of-function	Controversial	HCC patients	[157-160]
		c.121A>G/ c.122C>T/A	S	c	Thr41Ala/Ile/Asn	Gain-of-function	Controversial	HCC patients	[157-160]
		c.134C>G/A/T/c.133T>C/G	S	c	Ser45Cys/Tyr/Phe/Pro/Ala	Gain-of-function	Controversial	HCC patients	[157-160]
JAK1	JAK1	c.1932G>T/c.1933G>T (tandem mutation)	S	c	Gln644His/Val645Phe	Gain-of-function	ND	HCC *in vitro* and patients	[161]
		c.2108G>T	S	c	Ser703Ile	Gain-of-function	ND	HCC *in vitro* and patients	[161]
		c.2185A>T	S	c	Ser729Cys	Gain-of-function	ND	HCC *in vitro* and patients	[161]
KRAS	K-Ras	c.35G>T/A/c.34G>T/A	S	c	Gly12Val/Asp/Cys/Ser	Gain-of-function	Reduced survival	CCA patients	[162,132]
mtDNA	COX1	m.T6115C	S	c	Met71Thr	Loss-of-function	ND	HCC patients	[164]
	ATP8	m.G8387A	S	c	Val8Met	Loss-of-function	ND	HCC patients	[164]
	ND5	m.G13121A	S	c	Arg262His	Loss-of-function	ND	HCC patients	[164]
	ND6	m.T14180C	S	c	Tyr165Cys	Loss-of-function	ND	HCC patients	[164]
PIK3CA	PI3K p110α subunit	c.3204_320 5insA	S	c	Asn1068fs*4	Gain-of-function	ND	HCC patients	COSMIC
		c.3140A>G	S	c	His1047Arg	Gain-of-function	ND	HCC patients	COSMIC
		c.1624G>A	S	c	Glu542Lys	Gain-of-function	ND	HCC patients	COSMIC
		c.1633G>A	S	c	Glu545Lys	Gain-of-function	ND	HCC patients	COSMIC
PTEN	PTEN	Loss of hetero zygosity at 10q23	S	c		Lower expression	ND	HCC patients	[165,166]
TSC1	TSC1	c.2278delA	S	c	Arg760fs	Loss-of-function	ND	HCC patients	[167]
		c.965dupT	S	c	Met322fs	Loss-of-function	ND	HCC patients	[167]
TSC2	TSC2	c.3400G>A	S	c	Gly1134Ser	Loss-of-function	ND	HCC patients	[167]
		c.4653_4655 delAGA	S	c	1551_1552del	Loss-of-function	ND	HCC patients	[167]
		c.3050C>G	S	c	Thr1017Arg	Loss-of-function	ND	HCC patients	[167]
		c.2355G>T	S	c	Gln785His	Loss-of-function	ND	HCC patients	[167]
		c.4129C>T	S	c	Gln1377*	Loss-of-function	ND	HCC patients	[167]
		c.4129C>T	S	c	Gln1377*	Loss-of-function	Rapamycin sensitivity	HCC *in vitro* / HCC patients	[167]
		c.173C>T	S	c	Gln63*	Loss-of-function	Rapamycin sensitivity	HCC *in vitro* / HCC patients	[167]
		c.482-2A>T	S	nc	intron 5 splicing acceptor	Loss-of-function	ND	HCC patients	[167]
		c.2355+1G>T	S	nc	intron 21 splicing donor	Loss-of-function	ND	HCC patients	[167]
		c.1947-2delA	S	nc	intron 18 splicing acceptor	Loss-of-function	ND	HCC patients	[167]

Data obtained from COSMIC database, dbEMT and referred literature. CCA: cholangiocarcinoma; HCC: hepatocellular carcinoma; ND: not determined

#### Alteration in the expression and/or function of pro-apoptotic factors (MOC-5a)

The *TP53* gene encodes p53, which plays a key role as a tumor suppressor in several processes in response to cellular stress signals, regulating the transcription of many genes involved in cell cycle arrest, apoptosis, senescence, DNA repair and maintenance of genomic stability, among others. *TP53* is one of the most frequently mutated genes in HCC (25%-30%)^[140]^. Most of these mutations affect the DNA-binding domain of the protein, reducing its binding affinity to specific sequences of target genes. Cells harboring non-functional protein are less likely to induce apoptosis and, therefore, more resistant to DNA damage caused by chemotherapy^[141]^, which has clinical consequences in HCC patients^[142]^. A very common *TP53* missense mutation in HCC is c.747G>T (p.Arg249Ser), whose incidence has been related to exposure to aflatoxin^[143]^. In a study carried out in 409 HCC patients, c.747G>T (p.Arg249Ser) and c.469G>T (p.Val157Phe) mutations have been associated with poorer prognosis^[143]^. Another p53 mutation, c.743G>A (p.Arg248Gln), induces resistance to doxorubicin and paclitaxel in HCC. Cells harboring that mutation display enhanced expression of MDR1^[144]^, which is a known to be able to export both drugs.

Transcription factors related to p53, such as p63 and p73 are expressed as several isoforms due to alternative splicing. Although TAp63 and TAp73 isoforms are considered as tumor suppressors with pro-apoptotic activity^[145,146]^, N-terminal truncated isoforms, ΔNp63 and ΔNp73, display anti-apoptotic activity and stimulate proliferation. TAp63 and TAp73 down-regulation, and ΔNp63 and ΔNp73 overexpression have been found in HCC and they are related to shorter OS and tumor recurrence^[147]^. In addition, *in vitro* studies in HCC cells revealed that ΔNp63 isoform confers resistance to doxorubicin and mitoxantrone through the inhibition of factors involved in mitochondrial apoptosis pathways^[148]^.

*TP53* is also frequently mutated in CCA^[149]^. High expression of the truncated ΔN isoform Δ133p53, observed in CCA tissues, has been correlated with poor clinical outcome in patients suffering from this PLC^[150]^. Moreover, Δ133p53 isoform expression is increased in 5-FU-resistant CCA cells^[151]^. On the other hand, the presence of mutations in *TP53* and *CDKN2A* genes has been associated with poor prognosis in advanced CCA patients receiving a combination of gemcitabine and platinum-derived drugs as first-line therapy^[152]^.

Low levels of the pro-apoptotic factor TAp73 contribute to chemoresistance in CCA. Thus, TP73 expression is decreased in 5-FU-resistance CCA cell lines^[153]^. Deregulation of other pro-apoptotic proteins influences the response to anticancer drugs commonly used in CCA treatment. For example, decreased expression of Bax, which participates in the intrinsic apoptotic pathway, has been reported in gemcitabine-resistant cell lines^[156]^. However, Bax mutations have not been described in CCA samples.

Somatic mutations in several genes involved in the cell cycle regulation, including *CDKN2A* y *RB1*, have been identified. The presence of inactivating mutations in CDKN2A, a cyclin-dependent kinase inhibitor, has been associated with poorer prognosis in HCC^[119]^. In the case of *RB1*, a relationship between mutations in this gene and early recurrence of HCC after resection has been found^[155]^. Damaging mutations appearing in genes coding for other checkpoint proteins, which might be involved in carcinogenesis, have been identified^[119]^. However, no clinical consequences for these HCC patients have been reported. In contrast, a frameshift mutation in *CDKN1A* encoding a truncated protein which lacks the ability to interact with its targets has been found to confer resistance to paclitaxel in breast cancer cells^[168]^.

Caspase 8 plays a key role in signal transduction within the extrinsic apoptotic pathway. Somatic mutations with loss-of-function affecting this protein have been associated with the resistance to drugs whose mechanism of action includes apoptosis activation^[169]^. In a study of 69 HCC patients, 9 of them had the same alteration in the caspase 8 gene (*CASP8*), c.1225_1226delTG, a frameshift mutation with two base-pair deletion resulting in a defective protein with a shorter p10 protease subunit^[154]^. Mutations affecting p10 subunit of procaspase-8 have been reported to promote unresponsiveness to chemotherapy in other cancers, such as acute myeloid leukemia^[170]^. Whether these mutations are also involved in HCC chemoresistance is not known.

#### Alterations in anti-apoptotic/pro-survival factors (MOC-5b)

Aberrant expression and/or activating mutations in anti-apoptotic factors as well as constitutive activation of pro-survival signaling pathways, such as PI3K/AKT, Ras/Raf/MAPK/ERK/MEK or JAK/STAT, lead to an uncontrolled cell proliferation and evasion of apoptosis in cancer cells, which contributes to tumor progression and reduces effectiveness of chemotherapeutic drugs.

The PI3K/PTEN/AKT/mTOR pathway, commonly altered in HCC, is associated with poor prognosis^[171]^. The frequency of *PIK3CA* mutations in HCC is controversial, ranging from 0 to 36% of HCC cases depending on the population studied^[172,173]^. Some of the most recurrent *PIK3CA* mutations in HCC samples according to the data from COSMIC, such as c.3204_3205insA (p.Asn1068fs*4) and c.3140A>G (p.His1047Arg) are oncogenic^[174,175]^. Other mutations described in HCC, such as c.1624G>A (p.Glu542Lys) and c.1633G>A (p.Glu545Lys), affecting the PIK helical domain of the protein confer gain-of-function^[175]^. Although *PIK3CA* mutations have not been directly related to chemoresistance in HCC, an *in vitro* assay has reported *PIK3CA* overexpression in sorafenib-resistant HCC cells^[176]^.

The tumor suppressor gene *PTEN* is the major negative regulator of PI3K/AKT/mTOR pathway. Therefore, alterations leading to PTEN loss-of-function could induce the activation of this pathway. Even though PTEN mutations are uncommon in HCC, somatic loss of heterozygosity of PTEN allele has been found in 20%-30% of HCC cases^[165,166]^. Moreover, PTEN down-regulation may be also caused by epigenetic alterations^[177]^. These changes are clinically relevant because PTEN expression has been found to be decreased in sorafenib-resistant HCC cells^[176]^. Therefore, activation of PI3K/AKT/mTOR pathway due to impaired *PIK3CA* and *PTEN* genes may play a key role among MOC accounting for the lack of response of HCC patients to sorafenib. At this respect, several preclinical and clinical studies have been carried out to evaluate the efficacy of inhibitors targeting PI3K/PTEN/Akt/mTOR pathway. Some of them have shown promising results^[178]^. For instance, inactivating mutations in *TSC1/2* genes have been found both in HCC cell lines and clinical specimens, resulting in an impairment of mTOR signaling. However, HCC cells harboring these mutations were sensitive to rapamycin, an mTOR inhibitor^[167]^. TSC2-null HCC cell lines have also shown to be sensitive to everolimus, another mTOR inhibitor, and HCC patients with low expression of TSC2 treated with everolimus have higher OS rates^[179]^.

Regarding JAK/STAT signaling pathway in HCC tumors, somatic mutations mainly affecting domains of JAK1 (pseudo-kinase and tyrosine kinase) have been identified, which lead to constitutively activated JAK/STAT signaling^[161]^. Since JAK/STAT pathway is involved in acquired resistance of HCC cells to sorafenib^[180]^, these findings suggest that mutations in JAK1 may lead to failure of sorafenib treatment due to compensatory proliferation. Thus, mutated JAK1 could be a potential target for pharmacological manipulation. Indeed, cells harboring c.2108G>T (p.Ser703Ile) variant were sensitive to ruxolitinib, a JAK1/2 inhibitor^[181]^.

Wnt/β-catenin signaling pathway is frequently deregulated in HCC, leading to β-catenin accumulation in the nucleus of cancer cells^[159]^. Aberrant activation of this pathway is largely due to gain-of-function mutations in *CTNNB1* gene, encoding β-catenin protein, which have been observed in 20%-40% of HCC samples assayed^[182]^. These are somatic mutations usually located in exon 3 encoding the N-terminal phosphorylation sites of β-catenin. HCC sequencing studies collected in cBioportal database reveal that most frequent mutations occur in Ser/Thr phosphorylation residues (codons 33, 37, 41 and 45) and in codon 32. The clinical implication of these mutations is controversial. Some studies have associated the presence of *CNNTB1* mutations in HCC with better OS^[157,158]^, whereas others have linked these mutations to tumor progression and poor prognosis^[159,160]^.

The insulin-like growth factor (IGF) signaling cascade is also involved in cell growth and survival, and its activation plays an important role in the resistance of HCC to TKIs^[183,184]^. An *in vivo* assay has demonstrated the presence of elevated IG2F levels as one of the major mechanisms of acquired resistance to sorafenib in HCC^[184]^. In addition, it has been shown that c.747G>T (p.Arg249Ser) mutation in p53, which is very common in aflatoxin-induced HCC, is accompanied by enhanced expression of IGF2 and type 1 IGF receptor^[185]^. This suggests a possible link between this p53 mutation and the resistance of HCC to TKIs.

The presence of alterations in Ras/Raf/MEK/MAPK/ERK pathway may play an important role in the response of CCA to chemotherapy. Mutations in *KRAS* gene have been observed in different subtypes of CCA, mainly affecting codon 12, such as c.35G>T (p.Gly12Val), c.35G>A (p.Gly12Asp), c.34G>T (p.Gly12Cys) and c.34G>A (p.Gly12Ser), with variable incidence depending on the population under study^[162,186,187]^. These mutations have oncogenic potential, leading to constitutive stimulation of K-Ras and, consequently, activation of downstream signaling effectors^[188]^. Several studies have reported a reduced survival of CCA patients with mutations in K-Ras codon 12^[162,189]^. These mutations conferred resistance to everolimus in CCA cells^[163]^.

Both somatic mutations and reduced copy number of mitochondrial DNA (mtDNA) have been found in a large proportion of HCC tumors^[164,190]^. Some of these mutations affect coding regions and result in amino acid substitution or premature stop codon in polypeptides of respiratory complexes, which presumably leads to mitochondrial dysfunction. In tumor cells, this impairment results in altered reactive oxygen species (ROS) production, which can promote activation of survival pathways or changes in the expression of anti-apoptotic factors, eventually leading to an adverse impact on the response to chemotherapy. This is consistent with the finding that mtDNA depletion in HCC cells promotes resistance to 5-FU^[164]^.

## Novel mechanisms affecting chemotherapy efficacy

### Autophagy and changes in tumor microenvironment

Recent evidences have shown that tumor microenvironmental stress-induced autophagy may contribute in part to the development of chemoresistance^[191]^. Thus, in HCC cells treated with oxaliplatin autophagy is activated, which favors cell survival^[192]^. Moreover, oxygen deficiency triggers the activation of hypoxia-specific transcription factors, which regulates the expression of genes that increase cancer cell survival and drug resistance^[193]^. In addition, these factors are master regulators of the expression of genes involved in the phenotypic epithelial-mesenchymal transition (EMT), cell migration (*MMP2*), homing (*CXCR4*) and the establishment of the pre-metastatic niche (*LOX*)^[193]^. Somatic mutations in these genes have been described in HCC, although their relevance in protein function remains unknown [Table 9].

**Table 9:** Somatic (S) mutations affecting coding (c) and non-coding (nc) regions of genes related to tumor microenvironment in primary liver cancer

Gene	Protein	G/S	Region	Genetic mutations	Protein mutations	Functional consequences	Clinical consequences	Studies	References
IL6	IL6	S	c	c.179T>A	Ile60Asn	Moderate	Neutral	TCGA-LIHC	TCGA
		S	c	c.83C>T	Ala28Val	Moderate	Neutral	TCGA-LIHC	TCGA
		S	nc	c.20-6C>T	Splice region variant	Modifier	Neutral	TCGA-LIHC	TCGA
		S	nc	c.243+169T>G	Intron	Modifier	ND	TCGA-LIHC	TCGA
MMP2	MMP2	S	c	c.648G>T	Lys216Asn	Moderate	ND	TCGA-LIHC	TCGA
		S	c	c.1160C>G	Pro387Arg	Moderate	ND	TCGA-CHOL	TCGA
		S	c	c.85G>A	Ala29Thr	Moderate	Pathogenic	TCGA-LIHC	TCGA
		S	nc	c.-75-3345G>A	Intron	Modifier	Pathogenic	TCGA-LIHC	TCGA
CXCR4	CXCR4	S	c	c.664A>T	Ile226Phe	Moderate	Pathogenic	TCGA-LIHC	TCGA
		S	nc	c.-55C>A	5’UTR	Modifier	ND	TCGA-LIHC	TCGA
LOX	LOX	S	c	c.1144C>T	Pro382Ser	Moderate	Pathogenic	TCGA-LIHC	TCGA
		S	c	c.850T>A	Tyr284Asn	Moderate	Pathogenic	TCGA-LIHC	TCGA
		S	nc	c.*42T>A	3’UTR	Modifier	Pathogenic	TCGA-LIHC	TCGA

Data obtained from TCGA database. Functional consequences are based on VEP (Variant Effect Predictor; https://www.ensembl.org/vep) impact: High means that the variant is supposed to cause a high disruptive impact in the protein, which is likely to cause loss of function; Moderate means that the variant may be not disruptive, but results in a decrease effectiveness of the encoded protein; Modifier is usually referred to non-coding variants, whose impact is difficult to determine, although they can be involved in transcription or splicing changes. ND: not determined; TCGA: the cancer genome atlas; TCGA-LIHC: the cancer genome atlas - liver hepatocellular carcinoma; TCGA-CHOL: the cancer genome atlas - cholangiocarcinoma

In the case of CCA, interleukins, like IL-6, released by immune cells present in the tumor microenvironment, particularly macrophages, can confer resistance to toxic compounds and promote tumor growth. Targeting tumor microenvironment rather than CCA cells directly may lead to novel therapeutic strategies to treat this cancer^[194]^. In the case of HCC, higher expression of IL-6 also seems to be a key player. Moreover, IL-6 knockdown in HCC cells increased their sensitivity to sorafenib^[195]^. Whether several somatic mutations described in *IL-6* gene [Table 9] have any influence in MOC-6 of PLC is poorly understood.

### EMT-associated chemoresistance

EMT is the mechanism that leads to a transient and reversible de-differentiation of epithelial cells to a mesenchymal phenotype^[196]^. Changes occurred during EMT are evidenced by the loss of epithelial markers, such as E-cadherin^[197]^ and the increased expression of mesenchymal proteins such as N-cadherin, a-smooth muscle actin (a-SMA), fibroblast-specific protein (FSP-1) and EMT-transcription factors Snail (SNA1), Slug (SNA2), Twist and ZEB^[196]^. Among them, Snail is the most prominent inducer of EMT in HCC^[198]^. Although several somatic mutations in genes involved in EMT have been described [Table 10], the actual role in HCC chemoresistance of the resulting variants is not known.

**Table 10:** Somatic (S) mutations affecting coding (c) and non-coding (nc) regions of genes related to epithelial-mesenchymal transition (EMT) in primary liver cancer

Gene	Protein	Genetic mutations	G/S	Region	Protein mutations	Functional consequences	Clinical consequences	Studies	References
VIM	VIM	c.1024C>A	S	c	Arg342Ser	ND	ND	HCC patients	dbEMT
		c.1348A>G	S	c	Arg450Gly	ND	ND	HCC patients	dbEMT
SMAD3	SMAD3	c.425G>A	ND	c	Arg142His	ND	ND	HCC patients	dbEMT
HIF1A	HIF1A	c.984G>C	ND	c	Lys328Asn	ND	ND	HCC patients	dbEMT
TGFB1	TGFβ-1	c.528C>T	ND	c	Asn176Asn	ND	ND	Liver carcinoma	dbEMT
ZEB1	ZEB1	c.892G>C	S	c	Val298Leu	ND	ND	HCC patients	dbEMT
		c.777A>T	S	c	Leu259Phe	ND	ND	Liver carcinoma	dbEMT
		c.1219A>G	S	c	Ile407Val	ND	ND	Liver carcinoma	dbEMT
		c.824A>C	S	c	Lys275Thr	ND	ND	Liver carcinoma	dbEMT
ZEB2	ZEB2	c.80A>G	S	c	Asn27Ser	ND	ND	Liver Carcinoma	dbEMT
		c.1141A>G	ND	c	Met381Val	ND	ND	HCC patients	dbEMT
		c.1862T>C	ND	c	Val621Ala	ND	ND	Liver carcinoma	dbEMT
		c.855A>G	ND	c	Thr285Thr	ND	ND	Liver carcinoma	dbEMT
		c.2519G>T	ND	c	Ser840Ile	ND	ND	Liver carcinoma	dbEMT
CDH1	CDH1	c.884C>T	S	c	Thr295Ile	ND	ND	HCC patients	dbEMT
		c.1027C>T	S	c	Leu343Leu	ND	ND	Bile duct cancer	dbEMT
		c.1107C>T	S	c	Asn369Asn	ND	ND	Bile duct cancer	dbEMT
		c.900C>G	S	c	Ile300Met	ND	ND	Bile duct cancer	dbEMT
		c.1019C>T	S	c	Thr340Met	ND	ND	Bile duct cancer	dbEMT
		c.1070C>T	S	c	Thr357Ile	ND	ND	HCC patients	dbEMT
		c.925C>T	S	c	Pro309Ser	ND	ND	HCC patients	dbEMT
		c.427C>T	S	c	Pro143Ser	ND	ND	HCC patients	dbEMT
			S	c					
ILK	ILK	c.590C>A	S	c	Ser197Tyr	ND	ND	ND	dbEMT
		c.*1G>A	S	nc	3´UTR	Modifier	ND	TCGA-LIHC	TCGA
NES	Nestin	c.4489G>T	S	c	Gly1497Cys	Moderate	ND	TCGA-LIHC	TCGA
		c.2221C>A	S	c	His741Asn	Moderate	Neutral	TCGA-LIHC	TCGA
		c.2680T>A	S	c	Ser894Thr	Moderate	Neutral	TCGA-LIHC	TCGA
		c.3617G>T	S	c	Gly1206Val	Moderate	ND	TCGA-LIHC	TCGA
		c.4580G>A	S	c	Gly1527Asp	Moderate	ND	TCGA-LIHC	TCGA
		c.4569G>A	S	c	Met1523Ile	Moderate	Neutral	TCGA-LIHC	TCGA
		c.3770G>T	S	c	Gly1257Val	Moderate	ND	TCGA-LIHC	TCGA
		c.1176delC	S	c	Thr393fs*9	High	ND	TCGA-LIHC	TCGA
			S	c					
SNAI1	SNAI1	c.-8C>A	S	nc	5´UTR	Modifier	ND	TCGA-LIHC	TCGA
		c.*305A>G	S	nc	3´UTR	Modifier	ND	TCGA-CHOL	TCGA

Data obtained from TCGA and dbEMT databases. Functional consequences are based on VEP (Variant Effect Predictor; https://www.ensembl.org/vep) impact: High means that the variant is supposed to cause a high disruptive impact in the protein, which is likely to cause loss of function; Moderate means that the variant may be not disruptive, but results in a decrease effectiveness of the encoded protein; Modifier is usually referred to non-coding variants, whose impact is difficult to determine, although they can be involved in transcription or splicing changes. HCC: hepatocellular carcinoma; ND: non-determined; TCGA: the cancer genome atlas; TCGA-LIHC: the cancer genome atlas - liver hepatocellular carcinoma; TCGA-CHOL: the cancer genome atlas - cholangiocarcinoma

In healthy biliary epithelium, E-cadherin is located at the plasma membrane of cholangiocytes, whereas in malignant CCA cells down-regulation and cytoplasmic internalization of E-cadherin occurs. Mutations and epigenetic silencing by the hypermethylation of E-cadherin gene (CDH1) are some of the mechanisms accounting for its down-regulation, which correlates with poor tumor differentiation and metastasis^[199-204]^. Interestingly, CCA cells exhibiting mesenchymal traits are more resistant to gemcitabine than those characterized by a prominent epithelial phenotype^[205]^. In the case of HCC, the overexpression of nestin, a type VI intermediate filament protein, has been associated with EMT and chemoresistance^[206]^.

Alternative splicing may also affect EMT. The functional consequences of differential splicing in EMT is illustrated by p120 catenin, the adhesion protein cluster of differentiation 44 (CD44), and FGFR2. Many changes affecting alternative splicing during EMT come from the rapid down-regulation of two RNA-binding proteins: epithelial splicing regulatory protein 1 (ESRP1) and ESRP2. Their down-regulation results in the generation of pro-mesenchymal protein isoforms that lead to alterations in adhesion, motility and signaling pathways^[207-209]^.

## Conclusion

The information summarized in the present review clearly shows that germline and somatic mutations in genes involved in MOC play an important role in the overall response of HCC and CCA to chemotherapy. Although a remarkable advance in the identification and characterization of the functional consequences of these mutations has been achieved in the last decade it is evident that our current knowledge of this problem is still limited. This lack of information is partly due to the fact that most studies carried out so far on PLC chemoresistance have been focused on determining the expression levels of genes involved in MOC as well as their genetic and epigenetic regulation. Therefore, further investigations in this field are needed and highly recommended. Available information suggests that there is high probability of identifying, among genetic variants, both novel biomarkers to predict the failure of the pharmacological treatment and molecular targets to sensitize cancer cells to anticancer drugs, and hence improve the outcome of PLC patients.

## Declarations

### Copyright

© The Author(s) 2019.
